# Wetting-induced formation of void-free metal halide perovskite films by green ultrasonic spray coating for large-area mesoscopic perovskite solar cells†

**DOI:** 10.1039/d0ra07261c

**Published:** 2020-09-11

**Authors:** Sang Soo Kim, Jin Hyuck Heo, Sang Hyuk Im

**Affiliations:** a Department of Chemical and Biological Engineering, Korea University 145 Anam-ro, Seongbuk-gu Seoul 136-713 Republic of Korea imromy@korea.ac.kr

## Abstract

A void-free metal halide perovskite (MHP) layer on a mesoscopic TiO_2_ (m-TiO_2_) film was formed *via* the wetting-induced infiltration of MHP solution in the m-TiO_2_ film *via* a green ultrasonic spray coating process using a non-hazardous solvent. The systematic investigation of the behavior of ultrasonic-sprayed MHP micro-drops on the m-TiO_2_ film disclosed that the void-free MHP layer on the m-TiO_2_ film can be formed if the following conditions are satisfied: (1) the sprayed micro-drops are merged and wetted in the mesoscopic scaffold of the m-TiO_2_ film, (2) the MHP solution infiltrated into the m-TiO_2_ film by wetting is leveled to make a smooth wet MHP film, and (3) the smooth wet MHP film is promptly heat treated to eliminate dewetting and the coffee ring effect by convective flow in order to form a uniform void-free MHP layer. A void-free MHP layer on the m-TiO_2_ film was formed under optimal ultrasonic spray coating conditions of substrate temperature of ∼30 °C, spray flow rate of ∼11 mL h^−1^, nozzle to substrate distance of ∼8 cm, and MHP solution-concentration of ∼0.6 M under a fixed scan speed of 30 mm s^−1^ and purged N_2_ carrier gas pressure of 0.02 MPa. The mesoscopic MHP solar cells with an aperture area of 0.096, 1, 25, and 100 cm^2^ exhibited 17.14%, 16.03%, 12.93%, and 10.67% power conversion efficiency at 1 sun condition, respectively.

## Abstract 2

A void-free metal halide perovskite (MHP) layer on a mesoscopic TiO_2_ (m-TiO_2_) film was formed *via* the wetting-induced infiltration of MHP solution in the m-TiO_2_ film *via* a green ultrasonic spray coating process using a non-hazardous solvent.

## Graphical abstract

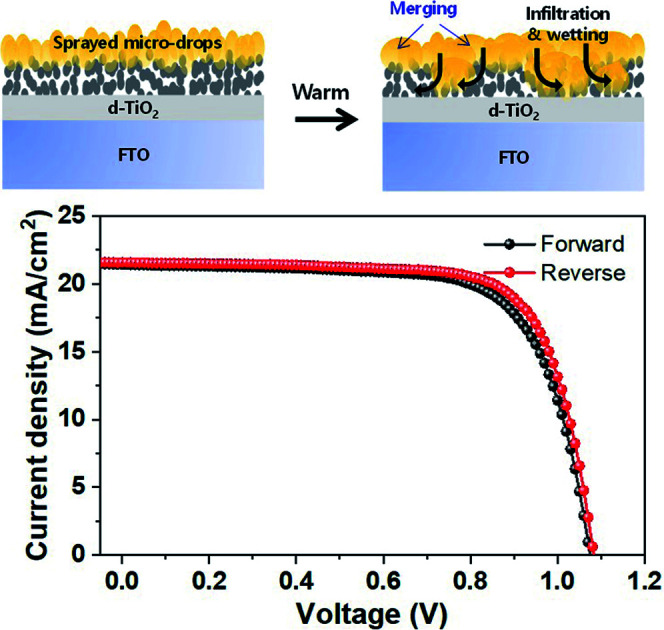

## Introduction

Since Grätzel *et al.* reported efficient dye-sensitized mesoscopic solar cells, mesoscopic TiO_2_ (m-TiO_2_) electrodes are being widely used because they not only provide a three-dimensional interconnected high surface area but also a co-continuous void space.^1^ Accordingly, m-TiO_2_ electrodes have been employed to fabricate efficient quantum dot-sensitized solar cells,^2–6^ metal chalcogenide-sensitized solar cells,^7–9^ and metal halide perovskite solar cells (MHP SCs).^10–14^

Among them, the MHP SCs such as CH_3_NH_3_PbX_3_ (MAPbX_3_: X = Cl, Br, I, and their mixture), CH(NH_2_)_2_PbX_3_ (FAPbX_3_), CsPbX_3_, and their mixtures have attracted significant attention because of their excellent properties such as strong absorptivity due to the direct bandgap, high open-circuit voltage due to the small exciton binding energy, convenient bandgap tunability by structure and composition engineering, and the mild/low temperature solution processability.^15^ Thus, similar to the above-mentioned sensitized solar cells, high performance MHP SCs with low current density–voltage (*J*–*V*) hysteresis with respect to the scan direction have been characterized by the mesoscopic device structure, which has a mesoscopic electron transporting layer (m-ETL) such as the m-TiO_2_.^16–18^

Besides the development of highly efficient MHP SCs, it is also important to develop a scalable coating process for the deposition of large-area MHP thin films for commercial applications as high-performance MHP SCs are fabricated *via* the spin coating process. Therefore, slot-die coating,^19,20^ blade coating,^21–23^ bar coating,^24–26^ spray coating,^27^ and ink-jet printing processes^28,29^ have been developed for the deposition of MHP thin films. Among them, the spray coating process is a unique process combining the advantage of a solution process and vapor deposition process and can form conformal MHP thin films even on rough surfaces with a stoichiometrically defined MHP solution. In addition, spray coating can also be used to form certain patterns by spraying MHP solution on a substrate with a patterned mask. Hence, the spray coating process, including air brush,^30–32^ ultrasonic spray,^33,34^ megasonic spray,^35^ and electrospray^36–38^ coating, has been studied intensively as a scalable process to replace the conventional spin coating process.

For instance, Heo *et al.* demonstrated 18.3% MAPbI_3−*x*_Cl_*x*_ MHP SCs composed of F-doped tin oxide (FTO)/dense TiO_2_ (d-TiO_2_)/MAPbI_3−*x*_Cl_*x*_/poly(triarylamine) (PTAA)/Au by depositing an MHP thin film *via* air brush coating.^32^ Park *et al.* reported 16.9% MAPbI_3_ MHP SCs constructed using indium tin oxide (ITO)/poly(3,4-ethylenedioxythiophene) polystyrene sulfonate (PEDOT:PSS)/MAPbI_3_/C_60_/bathocuproine (BCP)/Cu *via* the megasonic spray coating process.^35^ Su *et al.* fabricated 13.5% MAPbI_3−*x*_Cl_*x*_ MHP SCs composed of ITO/d-SnO_2_/MAPbI_3−*x*_Cl_*x*_/2,2′,7,7′-tetrakis[*N*,*N*-di(4-methoxyphenyl)amino]-9,9′-spirobifluorene (spiro-MeOTAD)/Au *via* an ultrasonic spray coating process under air blowing.^34^ Hong *et al.* demonstrated 13.27% ITO/PEDOT:PSS/MAPbI_3_/C_60_/BCP/LiF/Al *via* an electrospray coating process, which could control the size of the MHP solution droplets through the modulated applied electric field.^37^

Thus far, spray coating has been performed on dense flat substrates such as d-TiO_2_, d-SnO_2_, and PEDOT:PSS, which do not have any issue of pore filling because voids are often formed in the m-ETL/MHP layer due to the incomplete infiltration of MHP drops during the spray coating process.^38^ However, the m-ETL such as an m-TiO_2_ layer is important to reproducibly fabricate high performance MHP SCs with small current density–voltage (*J*–*V*) hysteresis because it prevents direct contact between the hole transporting material/Au and d-TiO_2_/FTO electrode. However, pinholes are formed in the MHP layer, which reduce the *J*–*V* hysteresis with respect to the scan direction due to the extended interface area balancing electron and hole flux, once the sprayed MHP micro-drops are wetted in the m-TiO_2_ ETL during the process. Recently, Heo *et al.* reported that the problem of void formation in m-TiO_2_ due to the incomplete infiltration of the MHP film during the electrospray coating process can be solved by using a vertically aligned TiO_2_ nanorod electrode with straight macropores.^38^ Therefore, systematic studies are still required to eliminate the voids in m-TiO_2_/MHP films due to the incomplete infiltration of the MHP solution drops during the spray coating process because the sprayed micro-drops are not easily wetted on and infiltrated in the m-TiO_2_ layer due to its three-dimensional interconnected nanopores.^38–40^

Herein, we used an ultrasonic spray coating process because it formed uniform MHP solution micro-drops. To achieve the perfect infiltration of the MHP solution micro-drops sprayed on top of the m-TiO_2_ ETL, we attempted to determine the optimum wetting conditions *via* systematic control of the ultrasonic spray processing parameters such as temperature, flow rate, nozzle height, and solution concentration. Consequently, we fabricated dense MHP thin films without voids *via* the wetting-induced perfect infiltration of the MHP solution micro-drops in the m-TiO_2_ ETL. In addition, we only used the non-hazardous γ-butyrolactone (GBL) solvent for the green ultrasonic spray coating process because the solvent in the atomized MHP solution micro-drops from the ultrasonic sprayer are more quickly evaporated than that in the bulk MHP solution, and thus it is desirable to avoid the use of hazardous solvents such as *N*-methyl-2-pyrrolidone, *N*,*N*-dimethylformamide, dimethylacetamide, and dimethyl sulfoxide.^41^

## Experimental

### Materials

Lead(ii) iodide (99% PbI_2_, Sigma-Aldrich), lead chloride (PbCl_2_, Sigma-Aldrich), hydroiodic acid (HI, 57 wt% in H_2_O distilled stabilized 99.95% HI, Sigma-Aldrich), hydrochloric acid (37 wt% HCl, Sigma-Aldrich), methylamine (TCI, 40 wt% in water), γ-butyrolactone (GBL, Sigma-Aldrich), acetonitrile (Sigma-Aldrich), toluene (Sigma-Aldrich), mesoscopic TiO_2_ paste (m-TiO_2_, Sharechem), titanium diisopropoxide bis(acetylacetonate) (Sigma-Aldrich), Li-bis(trifluoromethanesulfonyl)imide (Li-TFSI, Sigma-Aldrich), *tert*-butylpyridine (*t*-BP, Sigma-Aldrich), acetonitrile (Sigma-Aldrich), and poly(triarylamine) (PTAA, EM-index) were used as received.

### Preparation of MAI and MACl powder

Briefly, 35 mL of methylamine solution was charged in a round-bottom flask (RBF), which was immersed in an ice bath. Then, 50 mL of HI solution in a dropping funnel was slowly dropped into the RBF. After reacting for 1 h, the color of the solution changed to a yellowish color. The solution was magnetically stirred during the entire reaction. To collect the MAI powder, the product solution was dried using a rotary evaporator. To obtain pure MAI, the dried MAI powder was recrystallized by dissolution in ethanol and consecutive precipitation in diethyl ether. The recrystallized MAI powder was fully dried in a vacuum oven at 60 °C for 24 h. MACl was also synthesized following the above procedure except HCl solution was used instead of HI solution.

### Device fabrication

To fabricate mesoscopic MAPbI_3−*x*_Cl_*x*_ MHP SCs *via* the ultrasonic spray coating process, we deposited a 50 nm-thick d-TiO_2_ ETL on a patterned FTO (Pilkington, TEC7) substrate *via* the spray pyrolysis deposition of titanium diisopropoxide bis(acetylacetonate)/ethanol (1/9 vol/vol) solution at 500 °C with a hand brush (DH-125, Sparmax). Then an m-TiO_2_ paste/ethanol (1/5 wt/wt) solution was spin coated on the FTO/d-TiO_2_ substrate at 5000 rpm (revolution per min) for 30 s. The spin-coated FTO/d-TiO_2_/m-TiO_2_ film was then calcined at 500 °C for 1 h. For the deposition of the wetting-induced void-free MAPbI_3−*x*_Cl_*x*_ MHP film on the m-TiO_2_ layer, we prepared MAPbI_3−*x*_Cl_*x*_ MHP solutions by dissolving MAI : PbI_2_ : PbCl_2_ : MACl (1 : 0.95 : 0.05 : 0.1 mole ratio) powder in GBL solvent to obtain a specific concentration (0.2, 0.4, 0.6, 0.8, and 1.0 M). For the systematic studies to determine the wetting condition of MHP solution, we controlled the processing parameters such as substrate temperature (20 °C, 30 °C, and 60 °C), spray flow rate of MHP solution (7, 9, 11, 13, and 15 mL h^−1^), nozzle to substrate distance (4, 6, 8, 10, and 12 cm), and concentration of MHP solution (0.2, 0.4, 0.6, 0.8, and 1.0 M). Then, the MHP solution was deposited on the temperature-controlled FTO/d-TiO_2_/m-TiO_2_ substrate using an ultrasonic spray coater (S120, CERA-TORQ, 120 kHz) combined with a 3D printer (Creality, CR-10S Pro), where the MHP solution was delivered by a syringe pump (KD-Scientific, KDS100). After spraying the MHP solution, we waited for 25 s to ensure the wetting of the MHP solution on the m-TiO_2_ film and heat treated it on a hot plate pre-heated to 120 °C for 2 min. The scan speed of the ultrasonic sprayer and purged N_2_ carrier gas pressure for the sprayer was fixed to 30 mm s^−1^ and 0.02 MPa, respectively. We then spin coated a PTAA/toluene (15 mg mL^−1^) solution with 7.5 μL Li-TFSI/acetonitrile (170 mg mL^−1^) and 7.5 μL *t*-BP/acetonitrile (1 mL mL^−1^) on the FTO/d-TiO_2_/m-TiO_2_/MHP substrate at 3000 rpm for 30 s. Finally, the Au counter electrode was deposited on the FTO/d-TiO_2_/m-TiO_2_/MHP/PTAA substrate *via* thermal evaporation. The active area of small-sized MHP SC was fixed to 0.16 cm^2^. The large area MHP SCs were fabricated following our previous report.^42^ All experiments were conducted under an ambient air atmosphere and controlled relative humidity of ∼20%.

### Characterization

The SEM surface and cross-sectional images were measured using a high-resolution field emission scanning electron microscope (FE-SEM, FEI, Quanta 250, FEG). The external quantum efficiency (EQE) was measured using a power source (150 W xenon lamp, 13014, ABET) with a monochromator (MonoRa-500i, DongWoo Optron Co., Ltd.) and potentiostat (IviumStat, Ivium). The current density–voltage (*J*–*V*) curves were measured using a solar simulator (PEC-L01, Peccell) with a potentiostat (IviumStat, Ivium) under the illumination of 1 sun (100 mW cm^−2^ AM 1.5G), which was calibrated using a certified Si-reference cell (Japanese Industrial Standards). The *J*–*V* curves of the small MHP SCs were measured by masking the active area with a metal mask having an aperture of 0.096 cm^2^.

## Results and discussion

**Fig. 1:**
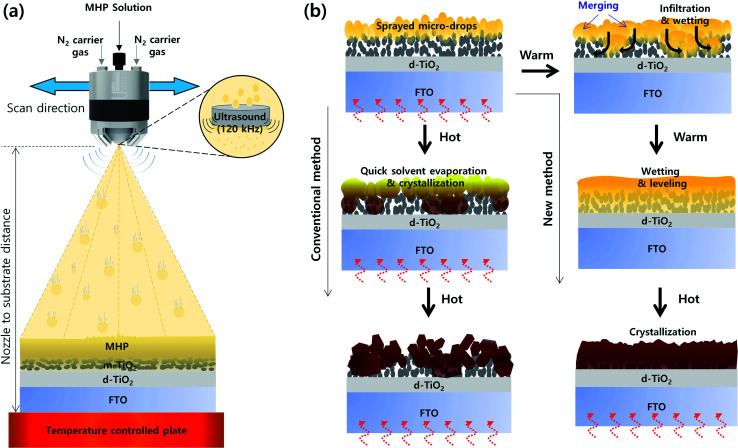
(a) Schematic structure of ultrasonic spray coater for the deposition of MHP solution and (b) schematic illustration of the morphologies of MHP/m-TiO_2_ films formed by the conventional and new wetting induced infiltration method.

Fig. 1(a) presents a schematic of the structure of the ultrasonic spray coater. The ultrasonic sprayer was connected to a 3D printer, which has a motorized *x*–*y* stage. Here, we deposited an MHP solution on an m-TiO_2_ film *via* single-pass spraying, and thus we only controlled the ultrasonic sprayer to the *x*-direction. The moving speed of the ultrasonic sprayer to the *x*-direction is defined to scan speed, which was fixed at 30 mm s^−1^. Accordingly, the ultrasonic spray coater can fabricate large-sized MHP solar sub-modules by multi-pass spraying. The MHP solution was fed to the ultrasonic sprayer by a syringe pump, and the feed (spray) flow rate was controlled to 7–15 mL h^−1^. The MHP solution in the nozzle end was atomized by 120 kHz ultrasound and the atomized MHP solution micro-drops were sprayed using N_2_ carrier gas whose pressure was fixed to 0.02 MPa. The sprayed volume of MHP solution per unit area of the m-TiO_2_ film could be controlled by the spray flow rate and the concentration of the MHP solution. The coating coverage and the sprayed volume of MHP solution per unit area could be simultaneously controlled by the nozzle to substrate distance. The evaporation rate of solvent in the MHP solution sprayed on the m-TiO_2_ film was controlled by the temperature of the plate.

Fig. 1(b) presents a schematic illustration of the morphologies of the MHP/m-TiO_2_ films formed *via* the conventional and new method. When the MHP solution is sprayed on the m-TiO_2_ film by the ultrasonic spray coater, initially the MHP micro-drops are positioned on top of the m-TiO_2_ film. If the m-TiO_2_ film is heated to a high temperature (∼120 °C), the MHP micro-drops on top of the m-TiO_2_ film will be quickly solidified by immediate solvent evaporation and crystallization occurs before perfect wetting and infiltration of the MHP solution into the m-TiO_2_ film. Therefore, some voids will be formed in the m-TiO_2_ film and a rough MHP film will be obtained. In contrast, if the m-TiO_2_ film is cold enough not to solidify the deposited MHP micro-drops by solvent evaporation, they will merge to form larger drops and simultaneously infiltrate the m-TiO_2_ film by wetting. With time, the MHP solution is perfectly infiltrated in the m-TiO_2_ film and spreads out by forming a smooth surface. At the time of perfect wetting of the MHP solution in the m-TiO_2_ film, if the solvent is quickly evaporated by heat treatment, a void-free dense MHP film can be obtained.

To confirm the formation of MHP micro-drops by the ultrasonic sprayer, we calculated the size of the MHP drops (*D*) formed by the ultrasonic sprayer using Lang's equation.^43^

1 *D* = 0.34(8π*σ*/*ρf*^2^)^1/3^

where *σ*, *ρ*, and *f* represent the surface tension of the MHP solution, density of the MHP solution, and ultrasound frequency (120 kHz), respectively. The *σ* of the MHP solution was calculated *via* the pendent drop method, as shown in Fig. S1.† The calculated drop sizes of the MHP solution formed by the ultrasonic sprayer were 9–15 μm. Therefore, we propose that the MHP micro-drops are deposited on the m-TiO_2_ film *via* the ultrasonic spray coater as illustrated in Fig. 1(b).

**Fig. 2:**
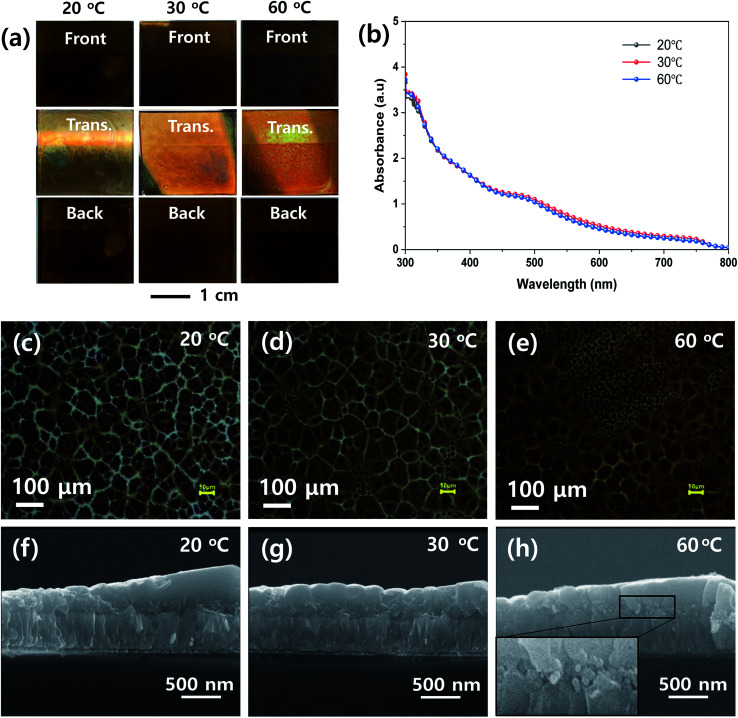
(a) Front, transmitted (Trans.), and back side photographs, (b) UV-vis absorption spectra, (c–e) optical microscopic images, and (f–h) cross-sectional scanning electron microscopy (SEM) images of MHP/m-TiO_2_ films formed by ultrasonic spray coater at different plate temperatures of 20 °C (c and f), 30 °C (d and g), and 60 °C (e and h), respectively.

To determine the proper processing parameters for the ultrasonic spray coating of MHP solution, we checked the effect of plate temperature on the wetting behavior, as shown in Fig. 2. The ultrasonic spray coating conditions are summarized in Table 1. Here, we deposited the MHP solution under a nozzle to substrate distance of 8 cm, spray flow rate of 11 mL h^−1^, and concentration of MHP solution of 0.6 M as a reference condition. The front, transmitted, and back side photographs of the FTO/d-TiO_2_/m-TiO_2_/MHP substrate formed by the ultrasonic spray coater at a plate temperature of 20 °C, 30 °C, and 60 °C are shown in Fig. 2(a). Apparently, the front and back side photographs of the 30 °C and 60 °C samples have uniform morphologies, whereas the 20 °C sample does not have a uniform morphology due to dewetting and coffee ring effect.^44^ The obtained photographs provide distinctive images of the coating uniformity. Although the coating uniformity of MHP film depends on the temperature of the plate, the UV-visible absorption spectra at different temperatures were similar, as shown in Fig. 2(b), because the mass of the deposited MHP film was same. The optical microscopy images in Fig. 2(c)–(e) indicate that the 30 °C sample has the largest MHP film domain size and the 60 °C sample has a non-uniform morphology due to the relatively quick evaporation of the GBL solvent. The corresponding scanning electron microscopy (SEM) cross-sectional images in Fig. 2(f)–(h) indicate that the 30 °C sample has an MHP film with a uniform thickness, whereas the others do not. For a better understanding of the pore filling of MHP in m-TiO_2_, the magnified SEM image is presented in the inset in Fig. 2(h). Unlike Fig. 2(f) and (g), Fig. 2(h) reveals that the m-TiO_2_ layer is not fully infiltrated with MHP because the sprayed micro-drops dried quickly. This imperfect infiltration of MHP in the m-TiO_2_ layer will seriously deteriorate the device efficiency because the generated electrons in MHP cannot be effectively transported to the m-TiO_2_ ETL. In addition, the uniform thickness of the MHP layer is also very important to obtain high efficiency. The uniformity of the film thickness is related to the wetting and dewetting phenomena and leveling of the sprayed drops. At high temperature, the sprayed drops dry quickly, and consequently form a rough MHP film. At a low temperature, the sprayed drops are fully infiltrated in the m-TiO_2_ layer *via* a wetting and leveling process, but the wetted MHP films are dewetted. Accordingly, a macroscopically non-uniform MHP layer is formed. To reveal the macroscopic and microscopic uniformity of the MHP films, we obtained photographs, optical microscopic images, and SEM images. Based on the above experimental results, subsequently we fixed the plate temperature to 30 °C because it resulted in the formation of the most uniform morphology in the MHP film.

**Table 1:** Summary of the MHP film uniformity with the processing parameters of ultrasonic spray coating (O: good, △: not bad, and x: not good)

Process parameter	Set value				
Plate temperature (°C)	20		30		60
	x		O		△
Spray flow rate (mL h^−1^)	7	9	11	13	15
	△	O	O	△	x
Nozzle to substrate distance (cm)	4	6	8	10	12
	x	△	O	△	x
MHP solution concentration (M)	0.2	0.4	0.6	0.8	1.0
	x	△	O	△	x
N_2_ carrier gas pressure (MPa)	0.02				
Scan rate (mm s^−1^)	30				

**Fig. 3:**
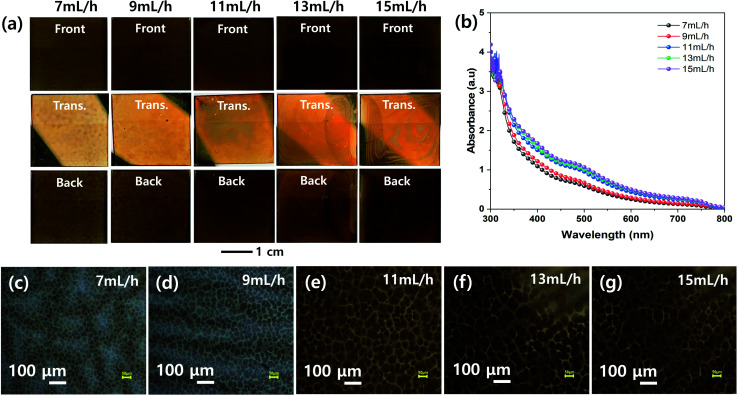
(a) Front, transmitted (Trans.), and back side photographs, (b) UV-vis absorption spectra, and (c–g) optical microscopic images of MHP/m-TiO_2_ films formed by ultrasonic spray coater at different solution flow rates of 7 (c), 9 (d), 11 (e), 13 (f), and 15 mL h^−1^ (g).

Fig. 3 shows the effect of spray flow rate on the morphologies of the MHP films during the ultrasonic spray coating process. The front, transmitted, and back-side photographs in Fig. 3(a) indicate that the uniformity of the ultrasonic-sprayed MHP film was good at a spray flow rate of 9–11 mL h^−1^, not bad at both 7 and 13 mL h^−1^, and not good at 15 mL h^−1^. The 7 mL h^−1^ sample had not sufficient volume of MHP solution to fully wet and infiltrate the m-TiO_2_ film, and thus some of the sprayed MHP micro-drops left spots upon drying. The 9–11 mL h^−1^ samples formed uniform MHP films because there was a sufficient volume of MHP solution to fully wet and infiltrate the m-TiO_2_ films. The 13–15 mL h^−1^ samples had non-uniform MHP films due to the inhomogeneous convective flow of the sprayed MHP solution by the Marangoni effect and coffee ring effect of the drying MHP solution *via* the pinning and depinning process. The UV-visible absorption spectra of the MHP/m-TiO_2_ films in Fig. 3(b) show that their absorptions gradually increased with an increase in the spray flow rate. The 11 mL h^−1^ sample had stronger absorptivity than the 7 mL h^−1^ sample, and thus the spray flow rate condition of 11 mL h^−1^ is desirable to make better MHP SCs. Their corresponding optical microscopy images in Fig. 3(c)–(g) indicate that the gradually intensified contrast with an increase in the flow rate is consistent with the absorption spectra and the domain sizes increased with the flow rate.

**Fig. 4:**
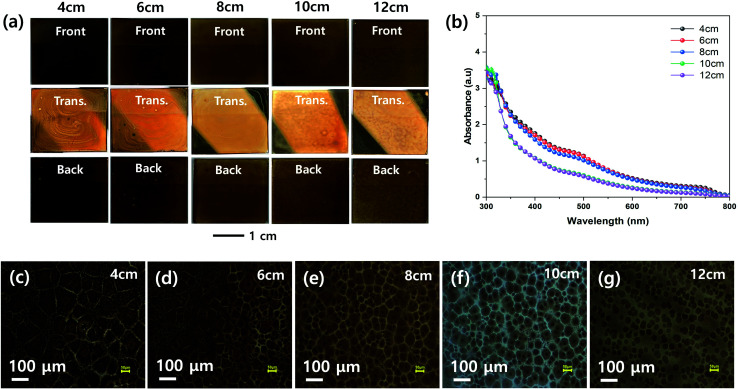
(a) Front, transmitted (Trans.), and back side photographs, (b) UV-vis absorption spectra, and (c–g) optical microscopic images of MHP/m-TiO_2_ films formed by ultrasonic spray coater at different nozzle to substrate distances of 4 (c), 6 (d), 8 (e), 10 (f), and 12 cm (g).

Fig. 4 shows the effect of the nozzle to substrate distance on the morphologies of the MHP films. The front, transmitted, and back-side photographs in Fig. 4(a) show that a uniform MHP film was obtained only with a nozzle to substrate distance of 8 cm. The MHP film uniformities of the 6 cm and 10 cm samples were not bad and the film uniformity of 4 cm and 12 cm samples were not good. These results imply that the MHP film uniformity is sensitive to the nozzle to substrate distance because the spray coated area is proportional to the square of the nozzle to substrate distance, and consequently the volume of sprayed MHP solution in a unit area has the same correlation. The UV-visible absorption spectra in Fig. 4(b) exhibit an inverse correlation between the nozzle to substrate distance and absorptivity. The corresponding optical microscopy images in Fig. 4(c)–(g) clearly indicate that the MHP domain sizes are significantly more dependent on the nozzle to substrate distance than the other processing parameters. Similar to the absorption spectra, the MHP domain sizes were inversely proportional to the nozzle to substrate distance.

**Fig. 5:**
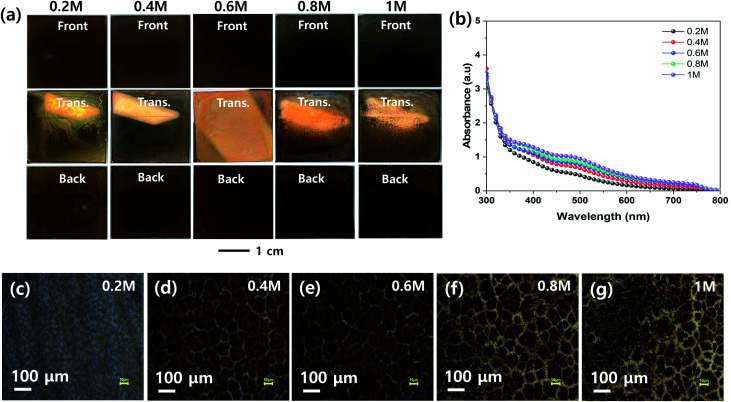
(a) Front, transmitted (Trans.), and back side photographs, (b) UV-vis absorption spectra, and (c–g) optical microscopic images of MHP/m-TiO_2_ films formed by ultrasonic spray coater at different MHP solution-concentrations of 0.2 (c), 0.4 (d), 0.6 (e), 0.8 (f), and 1.0 M (g).

The effect of the concentration of the MHP solution on the uniformities of the MHP films formed by the ultrasonic spray coating process was investigated, as shown in Fig. 5. The front, transmitted, and back-side photographs in Fig. 5(a) indicate that the 0.4, 0.6, and 0.6 M samples have relatively more uniform MHP films than the other samples. The 0.2 M sample had excess solvent, and consequently exhibited inhomogeneous convective flow patterns. The 1.0 M sample was quickly solidified because the sprayed MHP micro-drops were easily saturated by solvent evaporation and immediately crystallized before wetting the m-TiO_2_ film. Accordingly, it produced a rough MHP. The UV-visible absorption spectra in Fig. 5(b) indicate that the absorptions of the produced MHP films were linearly dependent on the concentration of the MHP solution in the ultrasonic spray coater. The corresponding optical microscopy images in Fig. 5(c)–(g) show that the domain sizes of the MHP films were gradually enlarged as the solution concentration increased from 0.2 M to 0.6 M, but were reduced at an MHP solution concentration of 0.8 M. The gradually intensified contrast implies that the thickness of the MHP film gradually increased with an increase in the concentration of the MHP solution. To confirm the morphologies and thickness of the MHP films produced by the ultrasonic spray coater with different concentrations of MHP solution, we measured their SEM surface and cross-sectional images, as shown in Fig. S2.† The thickness of the MHP film was ∼100 nm for 0.2 M, ∼200 nm for 0.4 M, ∼350 nm for 0.6 M, ∼500 nm for 0.8 M, and ∼750 nm for the 1.0 M sample.

**Fig. 6:**
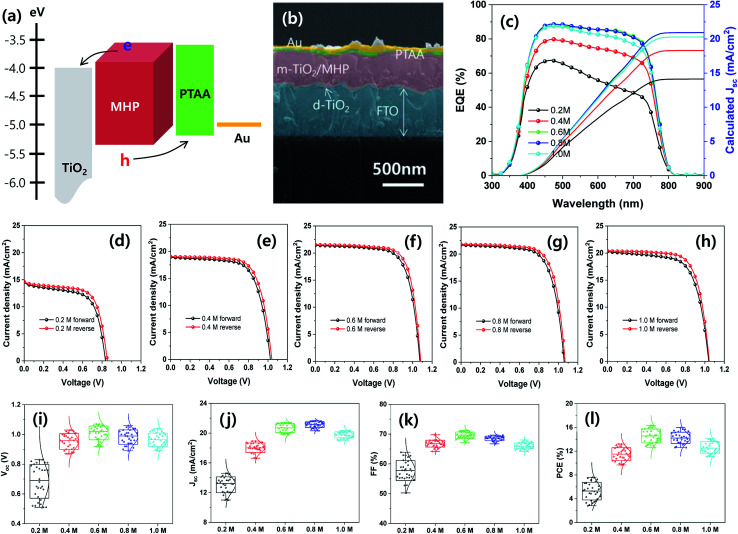
(a) Energy band diagram of MHP SC and (b) representative SEM cross-sectional image of MHP SC fabricated with 0.6 M MHP solution. (c) EQE spectra and calculated *J*_sc_ and (d–l) photovoltaic properties of MHP SCs prepared with different solution concentrations of MHP. (d–h) *J*–*V* curves of the best devices prepared with (d) 0.2, (e) 0.4, (f) 0.6, (g) 0.8, and (h) 1.0 M MHP solution and (i–l) average photovoltaic parameters of 30 samples for (i) *V*_oc_, (j) *J*_sc_, (k) FF, and (l) PCE.

Mesoscopic MAPbI_3−*x*_Cl_*x*_ MHP SCs composed of FTO/d-TiO_2_/m-TiO_2_/MHP/PTAA/Au by were fabricated by depositing the MHP thin films *via* the green ultrasonic spray coating process, as shown in Fig. 6. The energy band diagram and representative SEM cross-sectional image of the MHP SC fabricated with 0.6 M MHP solution are shown in Fig. 6(a) and (b), respectively. Upon illumination with light, the MHP film generates loosely bonded electron–hole pairs or free charge carriers due to its small exciton binding energy.^15,16^ The electrons are either injected into m-TiO_2_ or transported through the MHP layer, and are then transported to the d-TiO_2_/FTO electrode.^17^ Simultaneously, the holes are transferred/transported to the PTAA/Au electrode. By connecting the FTO and the Au electrode with an external circuit, the electrons continuously flow through the external circuit and recombine with holes by generating electricity.

The external quantum efficiency (EQE) spectra of the mesoscopic MHP SCs prepared with different MHP solution-concentrations of 0.2, 0.4, 0.6, 0.8, and 1.0 M are shown in Fig. 6(c). Apparently, the EQE values gradually increased as the concentration of the MHP solution increased from 0.2 to 0.6 M, and then the EQE values decreased with a further increase in the concentration of the MHP solution. The EQE value can be expressed as follows:

2 EQE = *η*_lhe_ × *η*_cs_ × *η*_cc_

where *η*_lhe_, *η*_cs_, and *η*_cc_ represent the light harvesting efficiency, charge separation (charge injection) efficiency, and charge collection efficiency, respectively. Thus, *η*_lhe_ monotonically increased with an increase in the concentration of the MHP solution, as shown in Fig. 5(b). Accordingly, the decrease in the EQE value for the 1.0 M sample can be attributed to the deteriorated *η*_cs_ and/or *η*_cc_. The morphologies of the MHP films prepared with different concentrations of MHP solution, as shown in Fig. 5 and S2,† indicate that the decline in the EQE value for the 1.0 M MHP solution concentration can be attributed to the formation of a relatively thick and rough MHP film on m-TiO_2_ owing to the quick drying of the solvent before complete wetting of the MHP solution during the ultrasonic spray coating process. The calculated short-circuit current density (*J*_sc_) values from the integration of the EQE spectra are 14.1 for 0.2 M, 18.3 for 0.4 M, 20.9 for 0.6 M, 20.9 for 0.8 M, and 20.3 mA cm^−2^ for the 1.0 M sample. The current density–voltage (*J*–*V*) curves of the corresponding MHP SCs with the best efficiencies, as shown in Fig. 6(d)–(h), indicate that the 0.6 M sample has the highest power conversion efficiency (PCE). The 0.6 M sample exhibited *J*_sc_ = 21.43 mA cm^−2^, open-circuit voltage (*V*_oc_) = 1.07 V, fill factor (FF) = 71.25%, and PCE = 16.34% for the forward scan condition and *J*_sc_ = 21.55 mA cm^−2^, *V*_oc_ = 1.08 V, FF = 73.64%, and PCE = 17.14% for the reverse scan condition. Therefore, the fabricated MHP SCs do not have significant *J*–*V* hysteresis with respect to the scan direction. The average photovoltaic parameters of 30 samples are shown in Fig. 6(i)–(l), and the average photovoltaic parameters of all the devices are summarized in Table 2.

**Table 2:** Summary of photovoltaic parameters of MHP SCs

Device	Concentration (M)	Scan direction	*J*_sc_ (mA cm^−2^)	*V*_oc_ (V)	FF (%)	PCE (%)
Aperture area (0.096 cm^2^)	0.2 (best)	Forward	14.61	0.83	62.86	7.62
		Reverse	14.66	0.85	66.38	8.27
	0.2 (average)	—	13.06 ± 1.07	0.68 ± 0.11	57.77 ± 3.37	5.28 ± 1.48
	0.4 (best)	Forward	18.93	1.03	67.72	13.20
		Reverse	19.05	1.04	70.02	13.87
	0.4 (average)	—	18.04 ± 0.64	0.95 ± 0.05	66.80 ± 1.16	11.50 ± 1.07
	0.6 (best)	Forward	21.43	1.07	71.25	16.34
		Reverse	21.55	1.08	73.64	17.14
	0.6 (average)	—	20.67 ± 0.60	1.01 ± 0.05	69.65 ± 1.06	14.57 ± 1.16
	0.8 (best)	Forward	21.68	1.06	69.47	15.96
		Reverse	21.81	1.07	71.85	16.77
	0.8 (average)	—	21.13 ± 0.39	0.98 ± 0.05	68.56 ± 0.77	14.24 ± 0.95
	1.0 (best)	Forward	20.28	1.04	66.92	14.11
		Reverse	20.43	1.05	70.32	15.08
	1.0 (average)	—	19.77 ± 0.40	0.97 ± 0.04	66.07 ± 1.01	12.62 ± 0.97
Aperture area (1 cm^2^)	0.6 (best)	Forward	21.45	1.07	69.85	16.03
		Reverse	21.51	1.08	72.29	16.79
	0.6 (average)	—	20.96 ± 0.47	1.04 ± 0.02	67.39 ± 0.02	14.64 ± 0.92
Aperture area (25 cm^2^)	0.6	—	6.54	3.03	65.28	12.93
Aperture area (100 cm^2^)	0.6	—	2.92	5.98	61.12	10.67

**Fig. 7:**
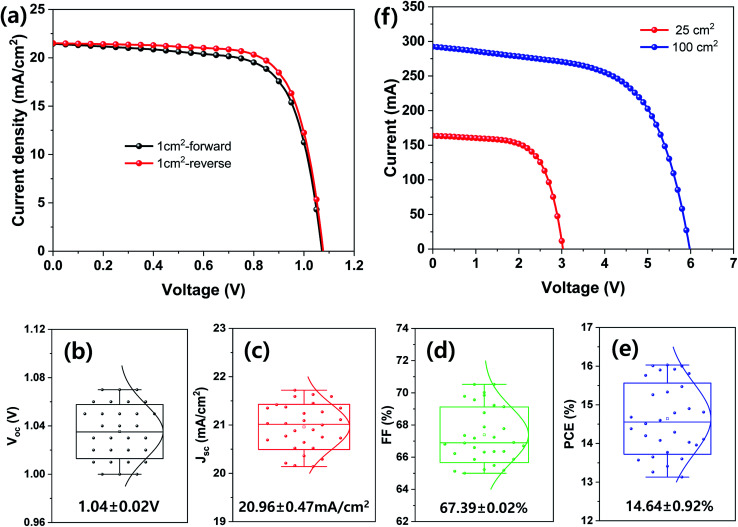
(a–e) Photovoltaic properties of MHP SCs with an aperture area of 1 cm^2^: (a) *J*–*V* curves of best device and (b–e) average photovoltaic parameters of 30 samples: (b) *V*_oc_, (c) *J*_sc_, (d) FF, and (e) PCE. (f) *J*–*V* curves of MHP SCs with an aperture area of 25 and 100 cm^2^.

Finally, large-area MHP SCs were fabricated and their photovoltaic properties are shown in Fig. 7. The best device PCE with an aperture area of 1 cm^2^ was 16.79% under 1 sun condition and the average *V*_oc_, *J*_sc_, FF, and PCE of the 30 samples were 1.04 ± 0.02 V, 20.96 ± 0.47 mA cm^−2^, 67.39% ± 0.02%, and 14.64% ± 0.92%, respectively. The MHP SCs with an aperture area of 25 and 100 cm^2^ exhibited a PCE of 12.93% and 10.67%, respectively.

## Conclusions

We successfully formed a void-free MHP layer on an m-TiO_2_ film *via* the wetting-induced perfect infiltration of MHP solution in the m-TiO_2_ film during the green ultrasonic spray coating process using the non-hazardous GBL solvent. To find the optimal process conditions for the formation of a void-free MHP layer on the m-TiO_2_ film, we controlled the substrate temperature, nozzle to substrate distance, and concentration of MHP solution under the fixed scan speed and N_2_ carrier gas pressure conditions of 30 mm s^−1^ and 0.02 MPa, respectively. Through systematic studies, we found that the possible processing windows of substrate temperature, spray flow rate, nozzle to substrate distance, and MHP solution-concentration for the formation void-free MHP films are 30–60 °C, 7–13 mL h^−1^, 6–12 cm, and 0.2–0.8 M, and the optimal process conditions are 30 °C, 11 mL h^−1^, 8 cm, and 0.6 M, respectively. Unlike the spin-coating process, the ultrasonic sprayer formed micron-sized MHP solution drops, and consequently the sprayed micro-drops on m-TiO_2_ film required wetting time for perfect infiltration of the MHP solution in the m-TiO_2_ film. For the wetting of the sprayed MHP micro-drops, the micro-drops should merge before solidification by quick solvent evaporation. Simultaneously, the wetted MHP micro-drops were leveled by forming a smooth surface. Here, the wetted film was heat-treated in order to quench the morphology of the wetted film. If the solvent is in excess in the wetted MHP film, the wetted film makes the MHP film non-uniform owing to the dewetting and coffee ring effect by convective flow during heat treatment. If the solvent is too low in the wetted MHP film, it quickly solidifies before leveling, and consequently produces a rough MHP film. Therefore, a void-free MHP layer on the m-TiO_2_ film could be obtained by optimizing the process conditions. Similarly the MHP SCs exhibited the best PCE of 17.14% under the optimal processing conditions. Using the optimal processing conditions, the large-area MHP SCs with an aperture area of 1, 25, and 100 cm^2^ exhibited a PCE of 16.03%, 12.93%, and 10.67%, respectively.

## Conflicts of interest

There are no conflicts to declare.

## Supplementary Material

RA-010-D0RA07261C-s001
