# Supplementary material for: Wetting-induced formation of void-free metal halide perovskite films by green ultrasonic spray coating for large-area mesoscopic perovskite solar cells
Source: RSC Adv. 2020 Sep 11;10(56):33651–61. doi: 10.1039/d0ra07261c (PMC9056762; doi:10.1039/d0ra07261c)
Supplement: RA-010-D0RA07261C-s001 [file RA-010-D0RA07261C-s001.pdf]

Electronic Supplementary Material (ESI) for RSC Advances.
This journal is © The Royal Society of Chemistry 2020

Electronic Supplementary Material

Wetting induced formation of void-free metal halide perovskite films by green ultrasonic spray coating for large-area mesoscopic perovskite solar cells

Sang Soo Kim,[a] Jin Hyuck Heo, [a] Sang Hyuk Im*,[a]

[a] Department of Chemical and Biological Engineering, Korea University, 145 Anam-ro, Seongbuk-gu, Seoul 136-713, Republic of Korea

**Corresponding authors:*

E-mail address: imromy@korea.ac.kr (S.H. Im)

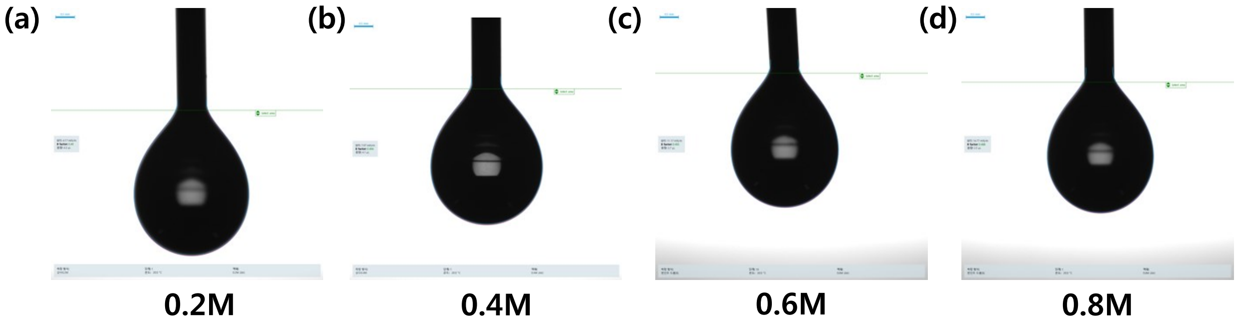

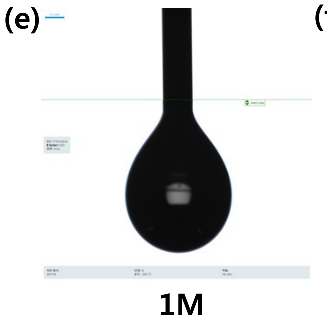

(f)

Concentration of MHP solution (M)	σ (mN/m)	ρ (g/mL)	D (μm)
0.2	4.17	0.124	9.15
0.4	7.97	0.249	11.37
0.6	11.17	0.373	12.89
0.8	14.77	0.500	13.89
1	17.50	0.621	14.76

Figure S1. (a-e) Photographs of pendant drops of MHP solutions with different concentration: (a) 0.2, (b) 0.4, (c) 0.6, (d) 0.8, and (e) 1 M; and (f) summary of corresponding solution's properties.

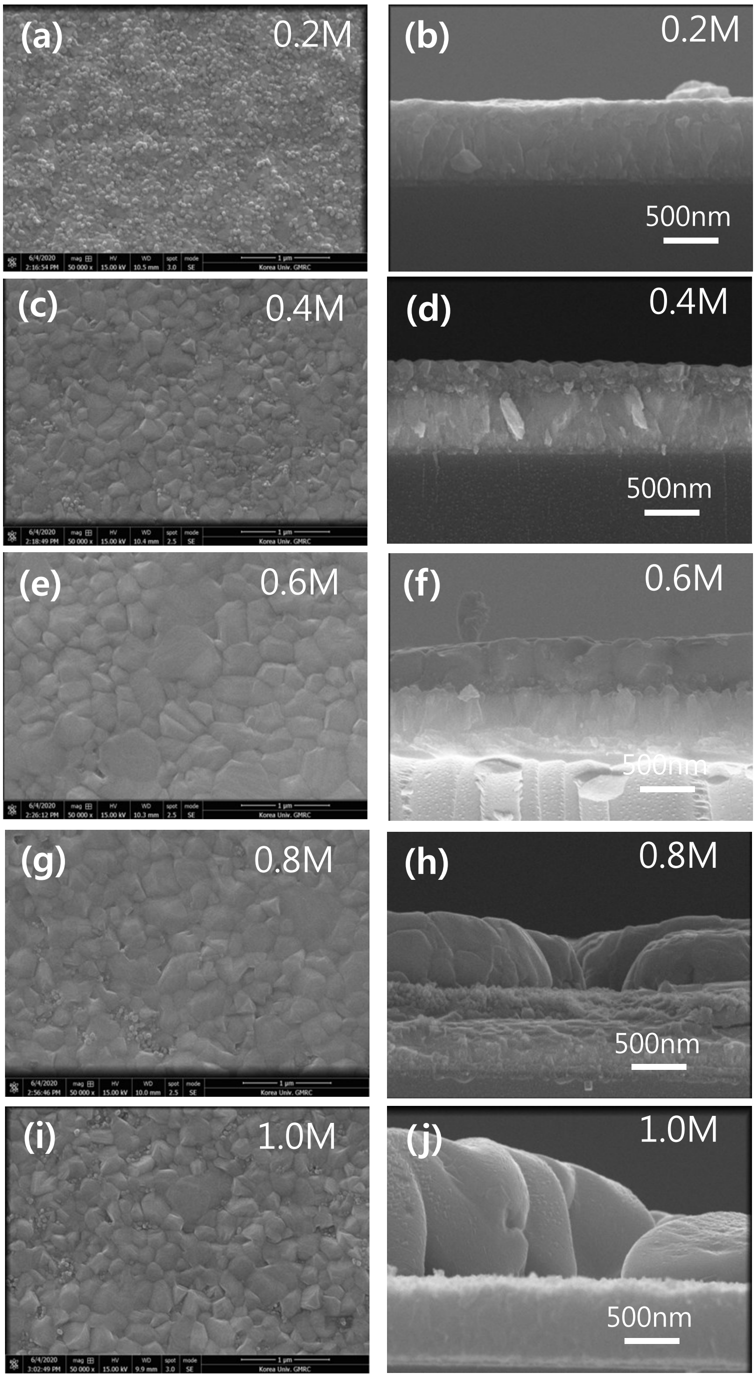

Figure S2. (a-j) SEM top surface (a, c, e, g, i) and cross-sectional images (b, d, f, h, j) of FTO/d-TiO_2/m-TiO_2/MHP prepared by different solution concentration: (a, b) 0.2, (c, d) 0.4, (e, f) 0.6, (g, h) 0.8, and (i, j) 1 M.
